# Circular RNA hsa_circ_0001658 regulates apoptosis and autophagy in gastric cancer through microRNA-182/Ras-related protein Rab-10 signaling axis

**DOI:** 10.1080/21655979.2021.2024637

**Published:** 2022-01-14

**Authors:** Xinxing Duan, Xiong Yu, Zhengrong Li

**Affiliations:** a Department of General Surgery, Affiliated Jiujiang Hospital of Nanchang University, Jiujiang, China; b Department of Gastrointestinal Surgery, The First Affiliated Hospital of Nanchang University, Nanchang, China

**Keywords:** Gastric cancer, circ_0001658, autophagy, apoptosis, miR-182, RAB10

## Abstract

Gastric cancer (GC) is a tumor with high incidence and lack of early diagnostic markers. The aim of this study was to explore novel regulatory circular RNAs (circRNAs) in GC and their underlying mechanisms. Differentially expressed circRNAs were analyzed using the Gene Expression Omnibus (GEO). mRNA and miRNA expression levels were determined using real-time reverse transcription polymerase chain reaction (RT-qPCR). Protein expression was detected using Western blotting. Cellular functions were evaluated using the cell counting kit-8 (CCK-8) assay and flow cytometry analysis. Immunofluorescence analysis was used to visually identify microtubule-associated protein 1 light chain 3 (LC3) puncta on a per-cell basis. Furthermore, dual-luciferase reporter and RNA pull-down assays were performed to verify the interaction between microRNA (miR)-182 and circ_0001658/Ras-related protein Rab-10 (RAB10). Circ_0001658 was identified to be aberrantly expressed in GC tissues and was demonstrated in GC cell lines (AGS and HGC27) *in vitro*. MiR-182 bound to circ_0001658 and RAB10. Circ_0001658 and RAB10 were upregulated, whereas miR-182 was suppressed in AGS and HGC27 cells. GC cell viability and autophagy were inhibited and apoptosis was promoted after circ_0001658 knockdown, and the cellular functions were reversed by downregulating miR-182. Moreover, upregulated RAB10 neutralized the effects of miR-182 on cell viability, autophagy, and apoptosis of GC cells. Silencing circ_0001658 restrained cell viability, suppressed autophagy, and promoted apoptosis of GC cells by sponging miR-182 to suppress the expression of RAB10. Therefore, circ_0001658 may be a potential therapeutic target for GC.

## Introduction

Gastric cancer (GC) is a prevalent malignancy and one of the top three cancers in terms of mortality worldwide [1,2]. Patients with GC are often diagnosed with advanced GC because of the lack of specific symptoms and diagnostic markers of early GC [3,4]. Although treatment methods such as molecular targeted therapy have emerged, surgical resection and adjuvant chemotherapy are still the main treatment methods for GC [5,6]. Due to chemotherapy drug resistance which leads to poor efficacy, an in-depth study on the mechanisms of occurrence and drug resistance of gastric cancer are of great significance in improving early diagnosis, prognosis, and survival rate of advanced gastric cancer.

The mechanisms and regulatory effects of circular RNAs (circRNAs) on the occurrence and development of GC have been extensively studied in the recent years, enriching the understanding of gene regulation under various pathophysiological conditions [7–9]. In addition, circRNAs have been reported as microRNA (miRNA) sponges, which are the most frequently reported roles of circRNAs, thereby affecting the cellular functions of cancer cells [10–12]. Meanwhile, miRNAs post-transcriptionally silence the target genes by binding to the 3ʹ untranslated region (3ʹUTR) of the messenger RNA (mRNA), thereby potentially modulating the biological processes [13,14]. Therefore, the circRNA-miRNA-mRNA network replenishes the understanding of competing endogenous RNA (ceRNA) regulatory mechanisms in various disorders, including GC [10,15–17]. Circ_0001658 is a novel circRNA located in the long arm of chromosome 6 (48,071 bp) and has been reported to promote the aggressiveness of osteosarcoma cells by sponging miR-382-5p to regulate YB-1 expression [18]. Currently, circ_0001658 has been identified to be abnormally expressed in GC tissues according to bioinformatics, suggesting the potential effects on GC progress [19].

Autophagy is a lysosomal-dependent protein degradation pathway that is widely present in eukaryotic cells to maintain intracellular homeostasis [20,21]. Various cells under stress conditions (such as radiotherapy and chemotherapy) will significantly increase the level of autophagy, and an imbalance in autophagy levels will lead to the onset of diseases such as tumors [22]. Therefore, autophagy is thought to be associated with tumors. Interestingly, autophagy has a bidirectional regulatory effect on GC development [23,24]. For instance, autophagy modulated by circCUL2 inhibits the deterioration of GC and enhances drug resistance [25]. Besides, it is well known that apoptosis is generally suppressed by autophagy, and autophagy is blocked by apoptosis-associated caspase activation [26,27]. Microtubule-associated protein 1 light chain 3 (LC3) and p62 have been identified as markers for monitoring autophagy and autophagy-related processes [22]. However, the underlying molecular mechanisms have not been fully elucidated yet.

In the present study, cell apoptosis and autophagy were investigated to understand the role of circ_0001658 in GC. Furthermore, the molecular mechanisms of circ_0001658 in the development of GC via sponging miR-182 to regulate Ras-related protein Rab-10 (RAB10) were also investigated in detail.

## Materials and methods

### CircRNA microarray analysis

Three microarray datasets of gastric cancer-related circRNAs (GSE93541, GSE89143, and GSE78092) were downloaded from the Gene Expression Omnibus (GEO) database. The R language limma package was used for standardized pretreatment and differentially expressed circular RNA (DEC) screening of the microarray data [28], and the Venn diagram of DEGs was drawn on the online website (http://bioinformatics.psb.ugent.be/webtools/Venn/). The downstream miRNAs of the differentially expressed circRNAs targeting as well as the target genes of miRNAs were predicted using starBase v2.0 (http://starbase.sysu.edu.cn/).

### Cell culture

The human gastric epithelial cell line GES-1 and human GC cell lines AGS and HGC27 were purchased from the Chinese Academy of Sciences (Shanghai, China). All cell lines were routinely cultured in DMEM (Gibco, NY, USA) medium containing 10% fetal bovine serum (Sigma, St. Louis, USA) at 37°C and 5% CO_2_. Cells were used for further experiments when they grew to 80–90% confluency.

### Plasmids and siRNA oligonucleotides

Small interfering RNA (siRNA) against circ_0001658 (si-circ_0001658 1#and si-circ_0001658 2#), siRNA negative control (si-nc), miR-182 mimic (mimic), miR-182 inhibitor (inhibitor), miRNA negative control (nc mimic and nc inhibitor), over-expressed RAB10 (oe-RAB10), and negative control (oe-nc) were generated by RiboBio (Guangzhou, China). Transfection was conducted in AGS and HGC27 cells with the constructed oligonucleotides (20 nM) or vectors (1 μg) via Lipofectamine 3000 (Thermo Fisher Scientific, CA, USA) as previously described [29]. Transfection validity was assessed using real-time reverse transcription polymerase chain reaction (RT-qPCR).

### RT-qPCR

RT-qPCR was used to detect the mRNA and miRNA levels. Total RNA was isolated from AGS and HGC27 cells using TRIzol reagent (Invitrogen, NY, USA). The cDNA was synthesized from 200 ng of extracted RNA using the Hi-Fi cDNA Synthesis Kit (Abcam, Cambridge, UK) and amplified by RT-qPCR using an ARIAMX real-time PCR system (Agilent, CA, USA). The target amplification process was performed as follows: pre-denaturation at 95°C for 5 min, denaturation at 95°C for 10s, annealing at 60°C for 20s, and extension at 72°C for 30s, for 40 cycles [28]. The relative expression levels of circ_0001658, miR-182, and RAB10 were calculated using the 2^−ΔΔCt^ method. All primers were synthesized and obtained from Shanghai Sangon Bitech (China). The primer sequences were as shown below: circ_0001658 (Forward, 5ʹ-GCCCAATCTCTCCTGCAAGT-3ʹ; Reverse, 5ʹ-CCACCTAGGAGGAACTGACAA-3ʹ); miR-182 (Forward, 5ʹ- UUUGGCAAUGGUAGAACUCACACU-3ʹ; Reverse, 5ʹ-UGUGAGUUCUACCAUUGCCAAAUU-3ʹ); RAB10 (Forward, 5ʹ-CACCGGATCGGGGATTCCGGAGTGG-3ʹ; Reverse, 5ʹ-AAACCCACTCCGGAATCCCCGATCC-3ʹ); *U6* (Forward, 5ʹ-GCUUCGGCAGCACAUAUACUAAAAU-3ʹ; Reverse, 5ʹ-CGCUUCACGAAUUUGCGUGUCAU-3ʹ), and *GAPDH* (Forward, 5ʹ- GGAGCGAGATCCCTCCAAAAT-3ʹ; Reverse, 5ʹ- GGCTGTTGTCATACTTCTCATGG −3ʹ).

### Cell viability assay

Cell viability was assessed using a Cell Counting Kit-8 kit (CCK-8, Doindo, Kumamoto, Japan). AGS and HGC27 cells were seeded in a 96-well plate (Thermo Fisher, CA, USA) with six replicates at a final concentration of 1 × 10^4^ cells/well. CCK8 solution (10 μL) and fresh medium (90 μL) were mixed and added to each well at 12, 24, 48, and 72 h [30]. Cell viability was detected at an absorbance of 450 nm (Synergy4; BioTek, Winooski, USA).

### Flow cytometry assay

Apoptotic cells were analyzed using flow cytometry (Beckman Coulter, CA, USA). AGS and HGC27 cells in each group were collected after transfection for 72 h, trypsinized, and washed twice with PBS after centrifugation. The cells that were incubated in binding buffer were stained with Annexin V and 7-AAD solution in the Apoptosis Detection Kit (Beyotime, Nantong, China). The apoptosis rate of the cells was detected using flow cytometry by analyzing the stained cells [30]. The samples were prepared in triplicate.

### Western blotting

AGS and HGC27 cells were washed with PBS and lysed in RIPA lysis buffer (Beyotime, Nantong, China). Protein concentration was determined using a BCA Protein Assay Kit (ab253410, Abcam, Cambridge, UK). After separation using 12% SDS-PAGE and transfer to a PVDF membrane, the proteins were sealed in 5% defatted milk powder for 2 h. Afterward, the diluted primary antibodies were added to the membranes, incubated overnight at 4°C, and then incubated with HRP-conjugated IgG (ab205718; 1/10,000) for 2 h. Finally, the protein band grayscale value was analyzed using Image Lab software (Bio-Rad, Hercules, USA). The primary antibodies used were obtained from Abcam (Cambridge, UK): anti-caspase-3 (ab32351, 1/5000), anti-BAX (ab32503, 1/1000), anti-Bcl-2 (ab32124, 1/1000), anti-cytosolic-associated protein light chain 3 (anti-LC3) (ab192890, 1/2000), and anti-p62 (ab91526, 0.5 µg/ml).

### Microtubule-associated protein 1 light chain 3 (LC3) Immunofluorescence Staining

AGS and HGC27 cells (10^5^ cells/well) were seeded into a 24-well plate (Thermo Fisher, CA, USA), fixed with 4% paraformaldehyde (Solarbio, Beijing, China) for 20 min, permeabilized with 0.1% Triton X-100 (Solarbio, Beijing, China) for 20 min, and then blocked with 4% bovine serum albumin (Gibco, CA, USA) for 1 h. AGS and HGC27 cells were incubated with anti-LC3 antibody (ab192890; Abcam, Cambridge, UK) at for 2 h, followed by a 30 min incubation with fluorescein isothiocyanate (FITC)-conjugated secondary antibody (ab6662; Abcam, Cambridge, UK) [31]. Subsequently, the cells were washed three times with PBS and fixed with a mounting medium (Solarbio, Beijing, China). Fluorescent images were acquired using a laser scanning confocal microscope (FV3000; Olympus, Beijing, China). ImageJ (National Institutes of Health, CA, USA) was used to establish methods to quantify the average number of LC3 puncta per cell in each group.

### Validation of targeting relationships

Dual luciferase reporter assays and RNA pull-down assays were conducted to verify the interactions between miR-182, RAB10, and circ_0001658 [32]. Wild-type luciferase reporter plasmids (circ_0001658-wt and RAB10 3UTR-wt) and corresponding mutant-types (circ_0001658-mut and RAB10 3UTR-mut) were transfected with the wild-type or mutant sequence of circ_0001658 or RAB10 3UTR containing miR-326 complementary sites into the pmirGLO luciferase reporter vectors (Promega, Madison, USA), respectively. Then, the miR-182 mimic or miR-NC plasmids were co-transfected into the generated luciferase reporter plasmids in AGS and HGC27 cells. Finally, the luciferase activity was analyzed in each group using a luciferase reporter assay kit (Promega, Madison, USA).

For the RNA pull-down assay (Pierce™ Magnetic RNA-Protein Pull-Down Kit, Thermo Fisher, CA, USA), the AGS and HGC27 cells were transfected with a biotinylated miR-182 mimic with the binding sequences between circ_0001658 or RAB10, and negative controls (NC mimic). The transfected cells of each group were lysed (RIPA lysis buffer, Yeasen, Shanghai, China) and incubated with probe-coated beads at 4°C for 3 h. Finally, the level of miR-182 was measured by using RT-qPCR.

## Statistical analysis

All data accessed from each experiment repeated in three independent experiments were expressed as mean ± standard deviation (SD) and analyzed using GraphPad Prism 8.3 software (GraphPad, San Diego, USA). Student’s t-test was used to analyze the differences between the two groups, and one-way analysis of variance (ANOVA) followed by Tukey’s test was used for multiple group analysis. Tukey’s test was used to verify the ANOVA for pairwise comparisons. Statistical significance was set at *P* < 0.05. Furthermore, the criterion for DECs in bioinformatics was |fold-change| ≥ 2. For quantification of LC3 puncta per cell, the average puncta count was determined by two-way ANOVA with Type III sum of squares.

## Results

### Circ_0001658 is identified to be up-regulated in GC

**Figure 1.**
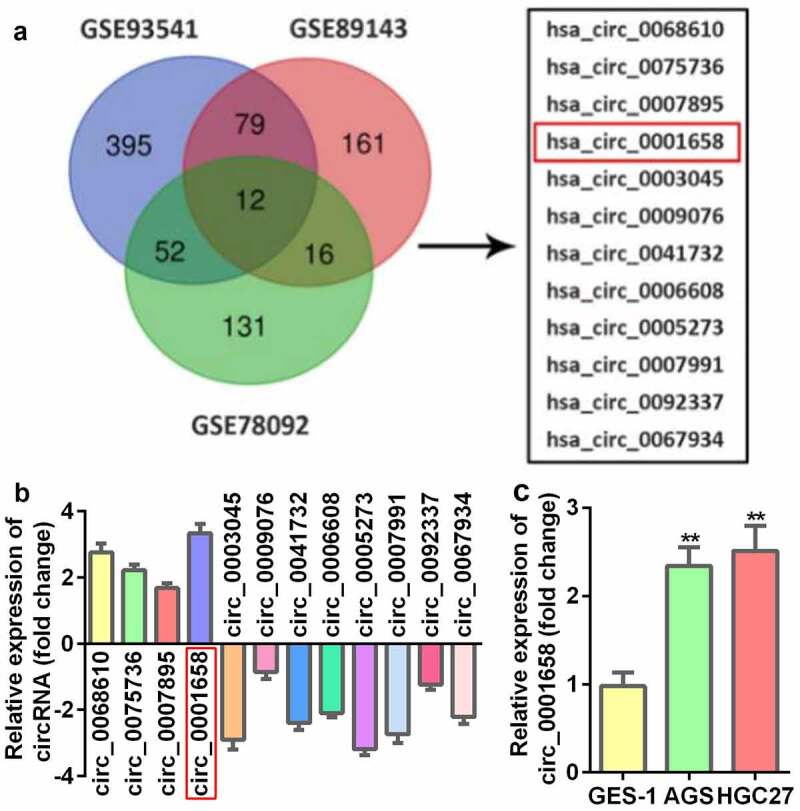
DECs in GC. (a) The venn diagram of DEGs. (b) The expression of DEGs measured by RT-qPCR. (c) The mRNA expression of circ_0001658 in GC cell lines. **P < 0.01, compared with GES-1 group.

A total of 12 circRNAs identified from three microarray data files were found to be abnormally expressed in GC tissues (Figure 1a), among which four were upregulated and eight were decreased (Figure 1b). In addition, the results of the bioinformatics analysis indicated that circ_0001658 was dramatically upregulated in GC tissues, which was verified in AGS and HGC27 cells (GC cells) compared with GES-1 cells (normal control) (**P < 0.01, Figure 1c).

### Down-regulated circ_0001658 suppresses cell viability as well as autophagy, and promotes cell apoptosis in GC cells

**Figure 2.**
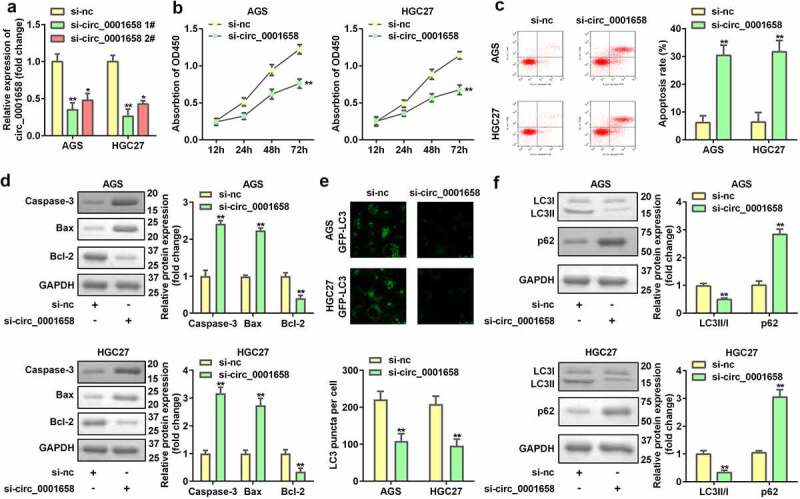
Down-regulated circ_0001658 regulated cellular functions of GC cells. (a) Circ_0001658 expression levels were detected using RT-qPCR after transfection. (b) Cell viability was detected using CCK-8. (c) Cell apoptosis was detected using flow cytometry. (d) Apoptosis-related proteins were measured by using Western blotting assay. (e) LC3 puncta were identified through immunofluorescence staining. (f) Autophagy-related proteins were measured using Western blotting assay. *P < 0.05, **P < 0.01, compared with si-nc group.

We evaluated the role of circ_0001658 on cellular fnction of GC cells. The expression of circ_0001658 in AGS and HGC27 cells was markedly downregulated by transfection with si-RNA, which was more remarkable after transfection with si-circ_0001658 2# plasmid (*P < 0.05, **P < 0.01, Figure 2a). After circ_0001658 was disrupted, cell viability was retarded, and cell apoptosis was facilitated (**P < 0.01, Figure 2b-2d). Furthermore, the decrease in LC3 puncta per cell of AGS and HGC27 cells induced by silencing circ_0001658 indicated that knockdown of circ_0001658 suppressed cell autophagy (**P < 0.01, Figure 2e), which was also reflected in the significant downregulation of LC3-II/LC3-I and upregulation of p62 at the protein level (**P < 0.01, figure 2f).

### miR-182 serves as a target of circ_0001658

**Figure 3.**
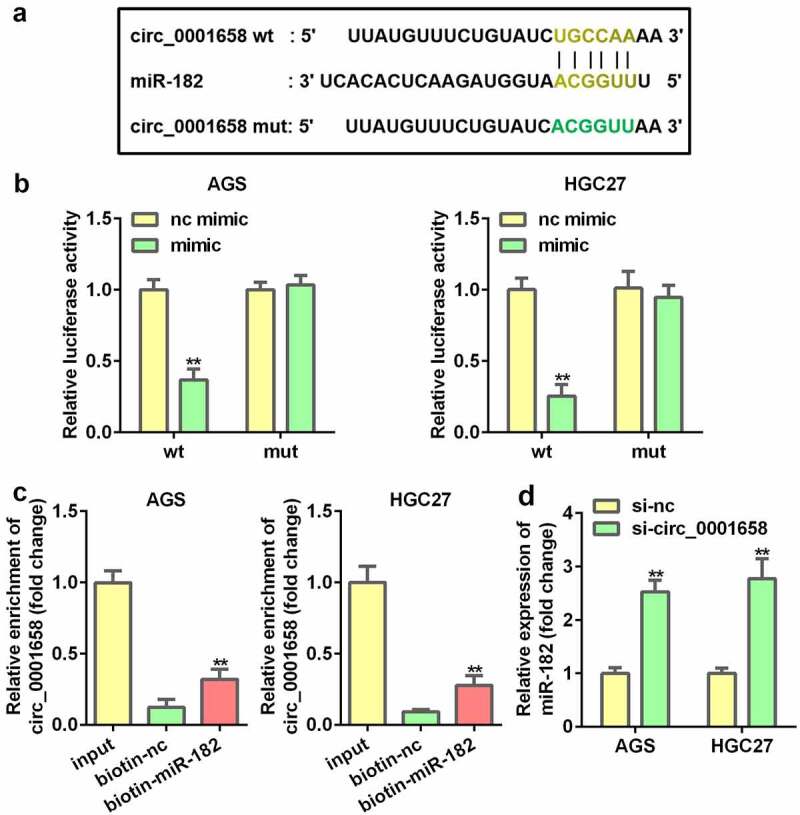
miR-182 was a target of circ_0001658. (a) Binding sites between miR-182 and circ_0001658 predicted by bioinformatics analysis. (b) Luciferase activity of AGS and HGC27 cells co-transfected with miR-182 mimic and wild-type or mutation type circ_0001658. (c) Enriched circ_0001658 levels of biotinylated miR-182 group and control group. (d) The expression of miR-182 was determined by using RT-qPCR. **P < 0.01, compared with nc mimic, biotin-nc, and si-nc group.

Interactions between miR-182 and circ_0001658 were verified. As shown in Figure 3a, the binding site predicted by bioinformatics analysis indicated that miR-182 is a target of circ_0001658. Luciferase assay results suggested that overexpression of miR-182 contributed to the notable reduction in luciferase activity of wild-type circ_0001658, whereas the luciferase activity in mutant circ_0001658 was invariant (**P < 0.01, Figure 3b), indicating that miR-182 can directly bind to circ_0001658. Moreover, circ_0001658 was enriched in biotin-miR-182, which further verified the interaction between miR-182 and circ_0001658 (Figure 3c). Furthermore, suppression of circ_0001658 led to the upregulation of miR-182 mRNA expression (Figure 3d).

### Inhibition of miR-182 abrogates the functions of suppressed circ_0001658 on cell viability, apoptosis and autophagy in GC Cells

**Figure 4.**
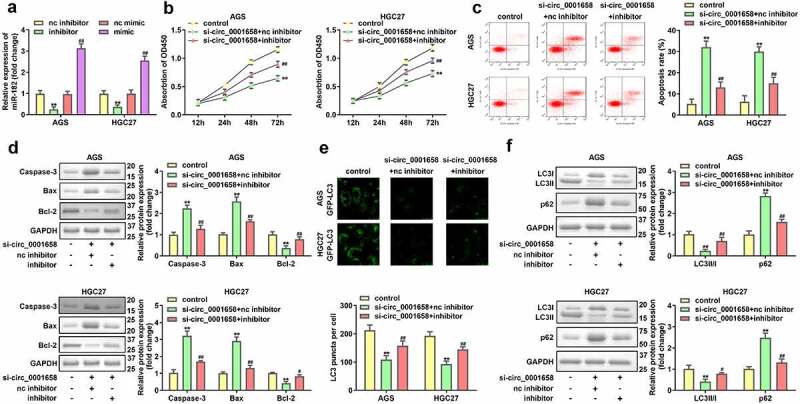
Effects of miR-182 on GC cells. (a) miR-182 expression levels were detected by using RT-qPCR after transfection. (b) Cell viability was detected using CCK-8. (c) Cell apoptosis was detected by using flow cytometry. (d) Apoptosis-related proteins were measured by using Western blotting assay. (e) LC3 puncta were identified through immunofluorescence staining. (f) Autophagy-related proteins were measured by using Western blotting assay. **P < 0.01, compared with nc inhibitor, and control group. #*P* < 0.05, ##*P* < 0.01, compared with nc mimic, and si-circ_0001658 + nc inhibitor group.

Then the effects of miR-182 on cellular fnction of GC cells were determined. PCR results showed that AGS and HGC27 cells were successfully transfected to significantly alter the expression levels of miR-182 in subsequent experiments (**P < 0.01, ##*P* < 0.01, Figure 4a). miR-182 interference notably restored circ_0001658 silence-mediated inhibition of viability, autophagy, and acceleration of apoptosis in AGS and HGC27 cells (**P < 0.01, #*P* < 0.05, ##*P* < 0.01, Figure 4b-4f).

### RAB10 is a target gene of miR-182

**Figure 5.**
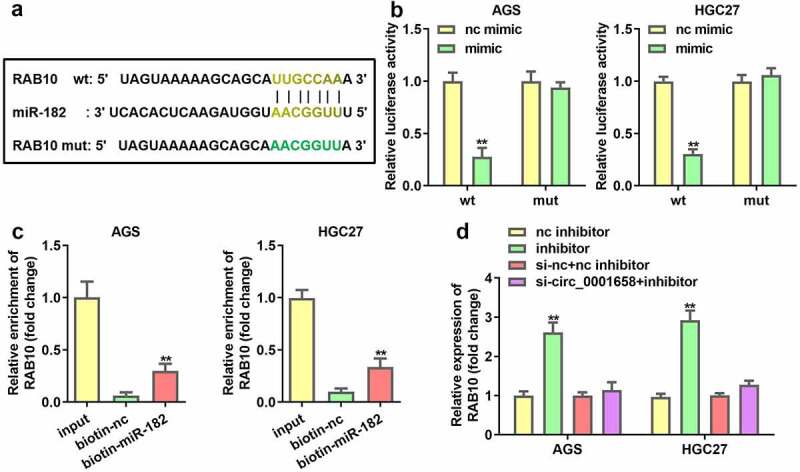
RAB10 was target gene of miR-182. (a) Binding sites between miR-182 and RAB10 predicted by bioinformatics analysis. (b) Luciferase activity of AGS and HGC27 cells co-transfected with miR-182 mimic and wild-type or mutation type RAB10. (c) Enriched RAB10 levels of biotinylated miR-182 group and control group. (d) The expression of RAB10 was determined by using RT-qPCR. **P < 0.01, compared with nc mimic, biotin-nc, and nc inhibitor group.

The seeding sites for miR-182 and RAB10 are shown in Figure 5a. A dual luciferase reporter and RNA pull-down assay was constructed to validate the prediction. The results demonstrated that luciferase activity in the wild-type RAB10 group was markedly reduced by miR-182 upregulation, while the activity in the mutant RAB10 group was not affected (**P < 0.01, Figure 5b). In addition, RAB10 was enriched in biotin-miR-182, which further verified the interaction between miR-182 and RAN10 (**P < 0.01, Figure 5c). In addition, the expression of RAB10 was significantly enhanced by the miR-182 inhibitor, which was abrogated by knockdown of circ_0001658 (**P < 0.01, Figure 5d).

### Over-expression of RAB10 inhibited the effects of up-regulated miR-182

**Figure 6.**
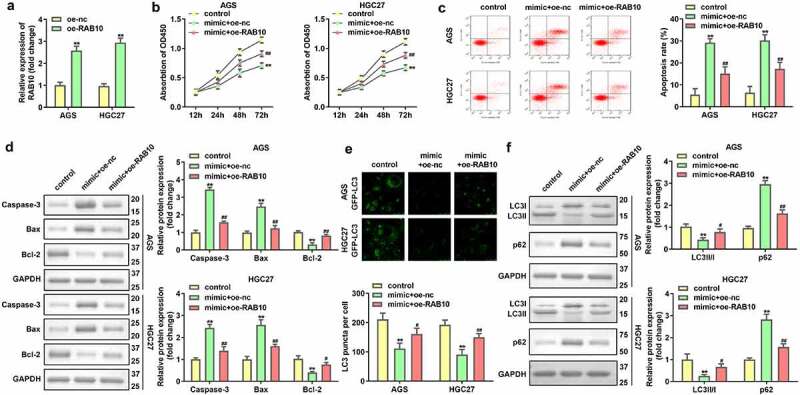
Effects of RAB10 on GC cells. (a) RAB10 expression levels were detected by using RT-qPCR after transfection. (b) Cell viability was detected by using CCK-8. (c) Cell apoptosis was detected by using flow cytometry. (d) Apoptosis-related proteins were measured using Western blotting assay. (e) LC3 puncta were identified through immunofluorescence staining. (f) Autophagy-related proteins were measured by using Western blotting assay. **P < 0.01, compared with oe-nc, and control group. #*P* < 0.05, ##*P* < 0.01, compared with mimic + oe-nc group.

RAB10 expression was significantly upregulated in AGS and HGC27 cells after transfection with RAB10 overexpression plasmids (**P < 0.01, Figure 6a). Upregulated miR-182-induced the inhibition of cell viability and promotion of cell apoptosis was attenuated by the overexpression of RAB10 (**P < 0.01, ##*P* < 0.01, Figure 6b). Furthermore, the increase in LC3 puncta per cell, LC3-II/LC3-I protein expression, and decrease in p62 protein expression in AGS and HGC27 cells co-transfected with miR-182 mimic and oe-RAB10 plasmids indicated that overexpression *of* RAB10 reversed the suppression of cell autophagy induced by upregulated miR-182 (**P < 0.01, #*P* < 0.05, ##*P* < 0.01, Figure 6e-f).

## Discussion

GC is one of the most common malignancies worldwide. It is critical to identify specific biomarkers to improve the early diagnosis and prognosis of GC [33]. Circ_0001658 has been shown to be aberrantly expressed in GC. Knockdown of circ_0001658 inhibited cell viability and autophagy, and promoted the apoptosis of AGS and HGC27 cells by sponging miR-182 to regulate RAB10.

Augmenting evidence reveals that abnormal expression of circRNAs in different malignancies can be used as diagnostic markers of tumors and targets for therapeutic intervention [34]. Zheng et al. found that circSEPT9 accelerated the tumorigenesis and development of triple-negative breast cancer [35]. CircSATB2 has been reported to facilitate the progression of non-small cell lung cancer [36]. Furthermore, dysregulated circRNAs, such as circSHKBP1 [12], circ_102958 [37], as well as circDONSON [38] motivated the occurrence and development of GC. Hence, circRNAs in GC with ectopic expression were suspected to be carcinogenic biomarkers. Our data suggested that circ_0001658 was aberrantly expressed in GC cells, and silencing circ_0001658 suppressed carcinogenesis by suppressing cell viability and autophagy, and promoting apoptosis.

It has been reported that circRNAs can serve as miRNA sponges to affect cellular functions to further regulate tumor development. In our study, miR-182 was found to be sponged by circ_0001658 in GC cells. miR-182 is an important regulator of malignant tumors because of its involvement in the initiation and progression of cancer [39]. miR-182 is also associated with distant metastasis in various cancer types and poor prognosis in patients [39]. Moreover, increasing evidence has demonstrated that miR-182 plays the role of a tumor suppressor in GC. For instance, Li et al. suggested that the deficiency of miR-182 reversed the inhibition of cell migration and invasion induced by circNRIP1 silencing in GC cells [40], which was in line with the research of Yu et al. on the role of miR-182 in GC [41]. In this study, silencing of circ_0001658 induced inhibition of cell viability, and autophagy was attenuated by miR-182 deficiency and promoted cell apoptosis.

RAB10 is a member of the Ras-related protein family and has been reported to function as an oncogenic gene in cervical cancer, hepatocellular carcinoma, esophageal squamous cell carcinoma, and osteosarcoma [42–45]. Interestingly, a growing body of evidence suggests that RAB10 plays a key role in regulating autophagy. For instance, RAB10 binds to the autophagy receptor optineurin to promote mitochondrial autophagy in Parkinson’s disease [46]. Meanwhile, RAB10 regulates autophagy in hepatocytes [47]. In the present study, RAB10 was shown to be a target gene of miR-182. Upregulated RAB10 alleviated the effects of miR-182 on cell viability, autophagy, and apoptosis.

## Conclusion

Our research suggests that circ_0001658 acts as a competing endogenous RNA to regulate the viability, autophagy, and apoptosis of GC cells via the miR-182/RAB10 axis. Knockdown of circ_0001658 may be an alternative treatment for GC.

## Highlights

- Down-regulated circ_0001658 regulated the cellular functions of GC cells.
- miR-182 was a target of circ_0001658.
- circ_0001658 regulates gastric cancer through miR-182/RAB10 signaling axis
